# Telomere length, genetic variants and risk of squamous cell carcinoma of the head and neck in Southeast Chinese

**DOI:** 10.1038/srep20675

**Published:** 2016-02-09

**Authors:** Yayun Gu, Chengxiao Yu, Limin Miao, Lihua Wang, Chongquan Xu, Wenjie Xue, Jiangbo Du, Hua Yuan, Juncheng Dai, Guangfu Jin, Zhibin Hu, Hongxia Ma, Hongbing Shen

**Affiliations:** 1 Department of Epidemiology, School of Public Health, Nanjing Medical University, Nanjing 211166, China; 2 Jiangsu Key Laboratory of Oral Diseases, Nanjing Medical University, Nanjing 210029, China; 3 Jiangsu Key Lab of Cancer Biomarkers, Prevention and Treatment, Collaborative Innovation Center for Cancer Personalized Medicine, Nanjing Medical University, Nanjing 211166, China

## Abstract

Telomere dysfunction participates in malignant transformation and tumorigenesis. Previous studies have explored the associations between telomere length (TL) and cancer susceptibility; however, the findings are inconclusive. The associations between genetic variants and TL have been verified by quite a few genome-wide association studies (GWAS). Yet, to date, there was no published study on the relationship between TL, related genetic variants and susceptibility to squamous cell carcinoma of the head and neck (SCCHN) in Chinese. Hence, we detected relative telomere length (RTL) by using quantitative PCR and genotyped seven selected single nucleotide polymorphisms by TaqMan allelic discrimination assay in 510 SCCHN cases and 913 controls in southeast Chinese. The results showed that RTL was significantly associated with SCCHN risk [(adjusted odds ratio (OR) = 1.19, 95% confidence interval (CI) = 1.08–1.32, *P* = 0.001]. Furthermore, among seven selected SNPs, only G allele of rs2736100 related to RTL in Caucasians was significantly associated with both the decreased RTL (*P* = 0.002) and the increased susceptibility to SCCHN in Chinese (additive model: adjusted OR = 1.17, 95%CI = 1.00–1.38, *P* = 0.049). These findings provide evidence that shortened TL is a risk factor for SCCHN, and genetic variants can contribute to both TL and the susceptibility to SCCHN in southeast Chinese population.

Telomeres consist of tandem (TTAGGG in humans)_n_ of nucleotide repeats at the ends of chromosome^1^, which have an important effect on protecting the end of chromosomes from atypical recombination, breakage, degradation, loss of nucleotides, and end-to-end fusion^2^,^3^,^4^. Human telomeres are 10-15 kb long and are approximately shorten by ~30 to 200 bp in each cycle of mitotic division^5^. Therefore, TL has been supposed to define the cellular life span and number of cell divisions^6^. While the telomeres turn into a critical threshold, they may lead to double-strand break and cell senescence or apoptosis^7^. It has been reported that telomere dysfunction plays a complex role in oncogenesis, and both very short and very long telomeres in peripheral blood lymphocytes (PBL) promote carcinogenesis^8^,^9^. Specially, an increasing number of epidemiologic studies have identified the associations between TL in PBL and risk of several cancer types, such as lung^10^,^11^,^12^,^13^, ovarian^14^, bladder^15^,^16^, breast^17^, and colon or rectum cancer^12^,^18^,^19^. Thus, TL in PBL may serve as a common marker of risk in human cancers.

Previous studies have indicated that telomere length is affected by plenty of factors, such as cigarette smoking^20^, oxidative stress^21^ and chronic inflammation^22^. Additionally, the variation of telomere length was also attributed to heritable factors^23^. For example, GWASs have verified that genetic variants in multiple genome regions and genes (3q26-*TERC*, 2p16.2-*ACYP2*, 4q32.2-*NAF1*, 19p12-*ZNF208*, 10q24.33-*OBFC1*, and 20q13.3-*RTEL1*) were associated with telomere length in European populations^24^,^25^,^26^,^27^. Meanwhile, researchers have identified that the genetic variants related to telomeres were associated with the risk of multiple cancers^11^,^24^,^25^.

The incidence of squamous cell carcinoma of the head and neck (SCCHN) ranks about the fifth among cancers^26^. An estimated 348,300 new cases and 179,600 deaths from oral and pharynx cancer in 2008 worldwide^27^. A few studies have evaluated the associations among telomere length, SNPs related to TL and SCCHN risk; however, all of them were conducted in European population and the results have been inconsistent. For example, a recent study showed that *TERT*-*CLPTM1L* variants were associated with both of the mean relative telomere length (RTL) and SCCHN risk in Icelandic and European populations^19^, but another study reported that telomere length and functional polymorphisms of *TERT* were not associated with risk of SCCHN in American population^28^. Up to date, no study has investigated the correlation between SNPs, telomere length, and SCCHN risk in Chinese population.

Therefore, in the present study, we designed a case-control study including 510 SCCHN cases and 913 cancer free controls in southeast Chinese to comprehensively investigate the associations between telomere length, SNPs related to RTL, and SCCHN risk in Chinese.

## Results

### Primary information

The distributions of age, gender and smoking status (*P* = 0.440, 0.056 and 0.890, respectively) showed no significant difference between 510 SCCHN cases and 913 controls (Table 1). However, there were more drinkers in cases than that in controls (49.90% vs 37.13%, *P* < 0.001). Among 510 cases, 403(79.02%) were with the oral cavity cancer, and 107 (20.98%) with others (Table 1). RTL was measured on DNA from blood samples, and t test showed that older people (age ≥ 60) had shorter telomere length than younger people (age < 60) (*P* = 3.21 × 10^−15^). Moreover, the liner regression analysis identified a significant correlation between TL and age (r = −0.118, *P* = 0.001) (Table 2 and Supplementary Figure 1). Besides, we found that RTL in male were significantly shorted than that in female (*P* = 0.01) (Table 2 and Supplementary Figure 2A), these results are consistent with the previous studies^29^,^30^,^31^. But no difference was found in subgroups with different drinking or smoking status (*P* > 0.05) (Table 2, Supplementary Figures 1 and 2A).

### The association between RTL and SCCHN risk

Then, we analyzed the associations between RTL and SCCHN risk and found that RTL in cases was significantly shorter than that in controls (OR = 1.19, 95%CI = 1.08–1.32, *P* = 0.001). In comparison with subjects with the first quarter (RTL ≥ 1.81), subjects with the third and fourth quarters had significantly increased risks of SCCHN with ORs (95%CIs) of 1.58(1.15–2.17) and 1.56(1.13–2.15), respectively (Table 3, Supplementary Figure 2B). When SCCHN cases were divided into two groups by tumor sites (oral cancer and other tumor sites), significant effect was only found for oral cancer (adjusted OR = 1.28, 95%CI = 1.14–1.43, *P* < 0.001), but not for other tumor sites (adjusted OR = 0.96, 95%CI = 0.80–1.15, *P* = 0.636), and the *P* value for heterogeneity test was 0.008 (Supplementary Table 1). Furthermore, the stratification analyses for the associations of RTL with SCCHN risk were conducted by age, gender, smoking and drinking status, and the results showed that the shorter RTL was associated with the increased risk of SCCHN in all subgroups except older (≥60). However, no significant heterogeneity was found among different strata (Supplementary Table 1).

### The effect of genetic variants on RTL

To evaluate the effect of genetic variants on RTL, we also analyzed the relevance between the seven SNPs reported in previous studies and RTL among 913 controls. Overall, we found that only the G allele of rs2736100 in *TERT* was significantly associated with the decreased RTL (*P* = 0.002), consistent with the findings in Caucasians^32^. But, we did not find any significant association for other six SNPs in controls (Table 4).

### The associations between genetic variants and SCCHN risk

Next, we evaluated the effect of promising SNP (rs2736100) on the susceptibility to SCCHN. Interestingly, we identified that G allele of rs2736100 was significantly associated with an increased risk of SCCHN (dominant model: adjusted OR = 1.11, 95%CI = 1.02–1.20, *P* = 0.013; additive model: adjusted OR = 1.17, 95%CI = 1.00–1.38, *P* = 0.049) (Table 5). In addition, analyses in different subgroup stratified by sex, gender, drinking and smoking status and tumor sites demonstrated the risk effect of rs2736100-G allele on SCCHN remained significant in subjects with old age (≥60), female, no-smoking, no-drinking status and oral cancers (*P* < 0.05). However, heterogeneity test demonstrated no significant difference among different strata (Supplementary Table 2).

## Discussion

It is the first case-control study to investigate the relationship between telomere length, genetic variants related to RTL and SCCHN risk in Chinese. We discovered that SCCHN cases, especially oral cancer cases, had shorter telomere length than controls. Meanwhile, our study identified that rs2736100 (*TERT* SNP) related to RTL in European was associated with both telomere length and SCCHN risk in this southeast Chinese population. Taking account of these findings, we speculate that genetic variants in *TERT* may affect the telomere length and consequently induce the altered risk of SCCHN.

**Figure 1:**
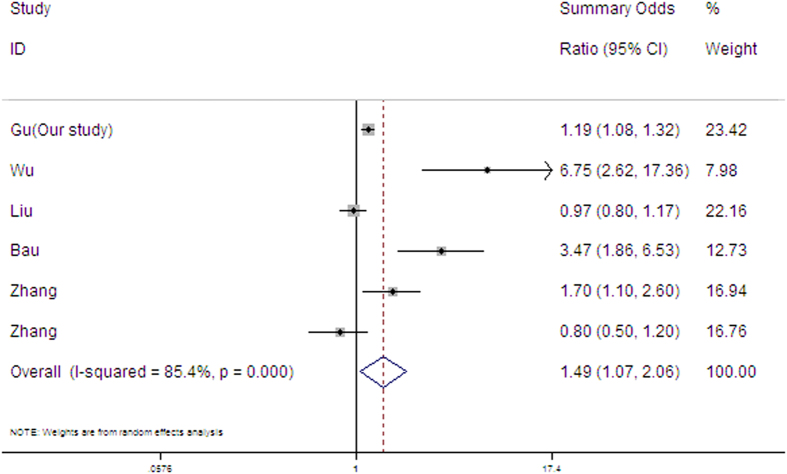
Meta-analysis of associations between telomere length and risk of SCCHN.

A few results have reported the relationship between RTL and SCCHN risk^33^,^34^,^35^,^36^, but the results are inconclusive because of different designs and sample size. We then performed a mini meta-analysis including our study and four available published studies on the association between RTL and SCCHN risk. The results of published studies were inconsistent with ORs of 6.75(2.62–17.36)^37^, 0.97(0.80–1.17)^28^, 3.47(1.86–6.53)^36^, 1.70(1.10–2.60)^33^ and 0.80(0.50–1.20)^33^. However, the pooled data showed that short RTL would significantly increase the risk of SCCHN (adjusted OR = 1.49, 95%CI = 1.07–2.06), supporting the findings in our study (Fig. 1 and Supplementary Table 3). Interestingly, our study also revealed a significant association between RTL and SCCHN in oral cancer, but not in other cancer sites, suggesting that telomere dysfunction might have different effects on the development of oral cancer and other HNSCC sites. However, these findings need the validation of other studies with larger samples and functional investigations.

Up to date, three GWAS studies have verified several SNPs related to RTL in European populations, including rs755017 at 20q13.33-*RTEL1*, rs10936599 at 3q26.2-*TERC*, rs4387287 at 10q24.33-*OBFC1*, rs7675998 at 4q32.2-*NAF1*, rs11125529 at 2p16.2-*ACYP2*, rs2736100 at 5p15.33-*TERT*, and rs8105767 at 19p12 -*ZNF208*^38^,^39^,^40^. In our study, we also discussed the association between above reported SNPs and RTL, and found that only rs2736100 in *TERT* was associated with RLT. *TERT* gene mapped to chromosome 5p15.33 encodes the telomerase enzyme and plays a key role in maintaining chromosomal stability and telomere DNA length^41^. Overexpression of *TERT* is likely to be involved in the tumorigenesis of multiple cancers including SCCHN^34^,^35^. The rs2736100 is located in intron 2 of *TERT* and the function of this SNP was still not clear. Although it is possible that this SNP has high linkage disequilibrium (LD) with other biologically plausible and cancer-causing mutations, a bioinformatics study indicated that rs2736100 may be localized in a regulatory region of the *hTERT* gene^42^. Thus, further functional investigations will be essential to deeply investigate the mechanism underlying these observations. Besides, we found no evidences of associations between other 6 SNPs and RTL, mostly because of genetic heterogeneity or small samples included in the published studies.

Recently, the relationship between genetic variants in *TERT* and the etiology of cancers has drawn increasing attention. Among those, rs2736100 is one of the most irrefutable identified SNP associated with cancer risk^42^,^43^,^44^. A recent meta-analysis including 16 published studies summarized the associations of rs2736100 with cancer risk and demonstrated that this SNP indeed affected the susceptibility to overall cancer^45^. However, no study has reported the role of rs2736100 in the development of SCCHN until now. In the present study, our results first identified that rs2736100 was associated with RTL and the altered risk of SCCHN in southeast Chinese population, providing more evidence that common genetic variants in *TERT* contribute to RTL and the carcinogenesis of SCCHN. Taking together, it may be plausible that rs2736100 can affect the *hTERT* expression, and subsequently generate a higher telomerase activity and an increased risk of SCCHN.

Additionally, it needs to be mentioned that RTL was significantly shorter both in the older (age ≥ 60) and in male, consistent with the findings in previous studies. For example, a lot of researches have confirmed that telomere length can act as a candidate biomarker of aging^46^,^47^,^48^,^49^. In additionally, several studies and a meta-analysis have identified that women have longer telomeres than men and such association would become stronger with increasing age^43^, which might result from a slower rate of telomere attrition in women^50^. Our study has several strengths. Up to now, it is the first study to systematically explore the associations among RTL, genetic variants related to TL and SCCHN risk in Chinese. Additionally, we recruited newly diagnosed cases and collected all blood samples prior to treatment, which may partly adjust the potential bias because of timing of blood draw for the case-control study. However, some insufficient points in our study also need to be improved. Firstly, we recruited SCCHN cases from hospitals and selected controls from communities, which might lead to potential selection bias. Second, a relative small sample size in this study might result in a lower statistical power. Third, we evaluated the RTL in peripheral blood leukocytes, but not in oral and neck tissue. However, some studies have investigated the consistence of TL between blood and tissues and showed significant linear correlation between leukocyte and some tissues^51^,^52^,^53^. Specially, Gadalla *et al.,* observed a significant correlation between blood and buccal cells measurements of RTL (r = 0.74, p < 0.0001)^52^. Finally, it is a retrospective case-control study and the blood samples were collected after cancer diagnosis, this limitation could potentially cause a reverse causation bias. Therefore, the findings of associations between RTL, rs2736100 and SCCHN risk in this study still require further large prospective studies which would carefully address potential bias and functional studies to elucidate the mechanisms underlying such associations.

## Materials and Methods

### Study subjects

This study was approved by the institutional review board of Nanjing Medical University and informed consent has obtained from all participants. SCCHN cases were constantly recruited from hospitals including Jiangsu Stomatological Hospital, Nanjing, China and the First Affiliated Hospital of Nanjing Medical University, since May 2009 to October 2013. All of the SCCHN cases were positive histology, and those undergoing radiotherapy or chemotherapy or having any history of tumor were removed from this study. Age (±5 years) and sex matched controls were randomly selected from a cohort including 30,000~ participants in a screening program based on community for non-infective diseases in Jiangsu, China. After knowing-agreeing, the interview was performed with a structured questionnaire to acquire general information and environmental exposure history, such as age, sex, ethnicity, smoking and alcohol drinking. After interview, ~5 ml venous blood sample were collected. Finally, 510 incident SCCHN cases and 913 frequency-matched controls with enough qualified DNA were included in this study. Participants who drank more than twice per week for longer than 1 year were defined as drinkers and those who smoked at least per day for more than 1 year were considered smokers.

### Measurement of relative telomere length

All methods and experimental protocols were approved by Nanjing Medical University, and carried out in accordance with the approved guidelines. GDNA was extracted from leukocyte of venous blood by phenol chloroform extraction. Each DNA sample was quantified and qualified by spectrophotometer and electrophoresis before genotyping. RTL was detected in genomic DNA using a modified RT-PCR protocol on an 7900HT(ABI PRISM) Sequence Detection System as we performed in previous study^54^. Telomere length was quantified by normalized the copy number of telomere repeats (T) to a single cope number (36B4, S). The reference pool contains equal amounts of genomic DNA from 5 healthy donors, to form a concentration related standard curve. The correlation coefficient (r^2^) of standard curve must be higher than 0.99. If the threshold cycle (Ct) value was out of the demarcation of the standard curves, the sample was repeated at a different concentration.

The q-PCR primers were shown in supplementary Table 4. Each well was poured into 50ng DNA pooled and 10μL SYBR^®^ Green (Applied Biosystems) PCR Master Mix. All samples for both the telomere and 36B4 reactions were carried out in duplicate. Laboratory technicians were blinded to the case-control status and equivalent controls and cases were tested in each well of 384-plate. Cawthon’s formula 2^−(ΔCt1−ΔCt2)^ = 2^−ΔΔCt^ was used to calculated RTL.

### SNP selection and genotyping

Up to date, three GWAS studies have discovered several genetic variants related to TL in European populations, such as rs398652, rs1317082 and so on^38^,^39^,^40^. However, no study has investigated the associations between these SNPs and TL or SCCHN risk in Chinese. According to above publications, we selected the reported SNPs with *P* ≤ 5 × 10^−8^ (genome-wide association significance level) and MAF ≥ 5% in Chinese population. After linkage disequilibrium (LD) analysis at r^2^ of 0.3, seven genetic variants related to telomere length were selected for genotyping, including rs755017 at 20q13.33-*RTEL1*, rs10936599 at 3q26.2-*TERC*, rs2736100 at 5p15.33-*TERT*, rs4387287 at 10q24.33*-OBFC1*, rs7675998 at 4q32.2-*NAF1*, rs11125529 at 2p16.2-*ACYP2*, and rs8105767 at 19p12-*ZNF208*. MAF and LD information were calculated by the 1000 Genomes Project (CHB&JPT subjects Phase I interim release).

The above SNPs were genotyped by means of the TaqMan allelic discrimination assay on the 7900HT (ABI PRISM Sequence Detection System). Supplementary Table 4 has shown the sequence of probes and primers. When genotyped, the lab technicians were blind with the subjects’ case or control status. Negative controls were added in every 384-plate and 10% of the samples were randomly selected to generate a 100% accordance. The yield rates of genotyping for the selected SNPs all reached 93% (Table 4).

### Statistical analysis

Data of genotyping and demographic characteristics of subjects between the cases and the controls were calculated by chi-square test. T-test was used to evaluate the distributions of relative telomere length in different status among controls. The goodness-of-fit chi-square test was used to test the HWE of all SNPs among the controls. Linear regression model was used to explore the relationship of single variant and telomere length among controls. Logistic regression model was used to evaluate the association between RTL or genetic variants and cancer risk. The heterogeneity of ORs and 95% CIs derived from relevant subgroups were tested by the meta-analysis. All statistical analyses were calculated with Stata 12.0.

Additionally, a mini-meta analysis was conducted by Stata version 12.0 using the “metan” code. Six case-control studies (peripheral blood leukocytes as DNA source) were included in this meta-analysis. ORs and 95%CI s of risk of RTL were collected to calculate the summary OR.

## Additional Information

**How to cite this article**: Gu, Y. *et al.* Telomere length, genetic variants and risk of squamous cell carcinoma of the head and neck in Southeast Chinese. *Sci. Rep.* **6**, 20675; doi: 10.1038/srep20675 (2016).

## Supplementary Material

Supplementary Information

## Figures and Tables

**Table 1:** Characteristics of the subjects in this study.

Variable	N (%)		*P*^a^
	Controls(n = 913)	Cases(n = 510)	
Age at diagnosis (years)			
Mean ± SD, y	59.78 ± 9.37	61.28 ± 10.72	0.440
<60	433(47.43)	231(45.29)	
≥60	480(52.57)	279(54.71)	
Sex			
Male	682(74.70)	357(70.00)	0.056
Female	231(25.30)	153(30.00)	
Smoking status^b^			
Never	451(49.40)	249(49.02)	0.890
Ever	462(50.60)	259(50.98)	
Drinking status^b^			
Never	574(62.87)	255(50.10)	0.001
Ever	339(37.13)	254(49.90)	
Tumor site			
Oral	−	403(79.02)	
Others	−	107(20.98)	

^a^Two-sided Chi-square test.

^b^The smoking and drinking status were unavailable for two and one controls, respectively.

**Table 2:** Distributions of relative telomere length (RTL) among controls.

Selected variables	N	RTL, mean(95%CI)	*P*^a^
Age at diagnosis (years)			
<60	433	1.61(1.58–1.66)	**3.21** × **10**^**−15**^
≥60	480	1.40(1.37–1.44)	
Sex			
Male	682	1.48(1.45–1.51)	**0.010**
Female	231	1.56(1.51–1.62)	
Smoking status			
Never	451	1.51(1.48–1.55)	0.569
Ever	462	1.50(1.46–1.53)	
Drinking status			
Never	574	1.52(1.48–1.55)	0.232
Ever	339	1.49(1.44–1.53)	

^a^Two-sided t test.

**Table 3:** Association between relative telomere length (RTL) and SCCHN risk.

Quartiles	RTL	Controls	Cases	Adjusted OR(95%CI)^a^	*P*
75%~	≥1.81	228(24.97)	98(19.22)	1	
50% ~ 75%	1.51–1.81	228(24.97)	98(19.22)	1.03(0.73–1.45)	0.864
25% ~ 50%	1.22–1.51	228(24.97)	157(30.78)	1.58(1.15–2.17)	**0.005**
~25%	<1.22	229(25.09)	157(30.78)	1.56(1.13–2.15)	**0.006**
Trend	−	−	−	1.19(1.08–1.32)	**0.001**

^a^Derived from logistic regression with an adjustment for age at blood collection, sex, smoking and drinking status.

**Table 4:** Associations of the reported loci with relative telomere length (RTL) among controls.

Loci	Chr.	Gene	Alleles^a^	Call rate (%)	MAF^b^	β^c^	*P*^c^
rs10936599	3q26.2	*TERC*	T/C	98.1	0.465	−0.022	0.370
rs11125529	2p16.2	*ACYP2*	C/A	98.6	0.214	0	0.991
rs2736100	5p15.33	*TERT*	T/G	98.2	0.419	−0.060	**0.002**
rs4387287	10q24.33	*OBFC1*	A/C	96.1	0.160	−0.002	0.956
rs755017	20q13.33	*RTEL1*	A/G	93.8	0.443	−0.002	0.924
rs7675998	4q32.2	*NAF1*	A/G	98.4	0.187	−0.029	0.356
rs8105767	19p12	*ZNF208*	A/G	98.5	0.289	−0.046	0.088

^a^Effect allele/alternative allele; effect allele is one associated with short telomeres, corresponding to the negative value of β estimates.

^b^Minor allele frequency among controls in this study.

^c^Derived from generalized linear models with an adjustment for age at blood collection and sex.

**Table 5:** Associations between rs2736100 and SCCHN risk.

Gene	SNP	Controls	Cases^a^	Adjusted OR (95%CI)^b^	*P*^b^
		N (%)	N (%)		
*TERT*	rs2736100	897	495		
	TT	317(35.3)	144(29.1)	1	
	TG	419(46.7)	255(51.5)	1.37(1.06–1.78)	**0.015**
	GG	161(18.0)	96(19.4)	1.32(0.95–1.83)	0.099
	TG + GG	580(64.7)	351(70.9)	1.11(1.02–1.20)	**0.013**
	Additive model			1.17(1.00–1.38)	**0.049**

^a^Genotypes were available from 495 cases and 897 controls

^b^Derived from logistic regression with an adjustment for age, sex, smoking and drinking status.
