# Supplementary material for: Telomere length, genetic variants and risk of squamous cell carcinoma of the head and neck in Southeast Chinese
Source: Sci Rep. 2016 Feb 9;6:20675. doi: 10.1038/srep20675 (PMC4746643; doi:10.1038/srep20675)
Supplement: Supplementary Information [file srep20675-s1.docx]

# Telomere length, genetic variants and risk of squamous cell carcinoma of the head and neck in Southeast Chinese

# Yayun Gu^1†^, Chengxiao Yu^1†^, Limin Miao^2^, Lihua Wang^1^, Chongquan Xu^1^, Wenjie Xue^1^, Jiangbo Du^1^, Hua Yuan^1^, Juncheng Dai^1,3^, Guangfu Jin^1,3^, Zhibin Hu^1,3^, Hongxia Ma^1,3*^, Hongbing Shen^1,3*^

**Supplementary Figure 1:**


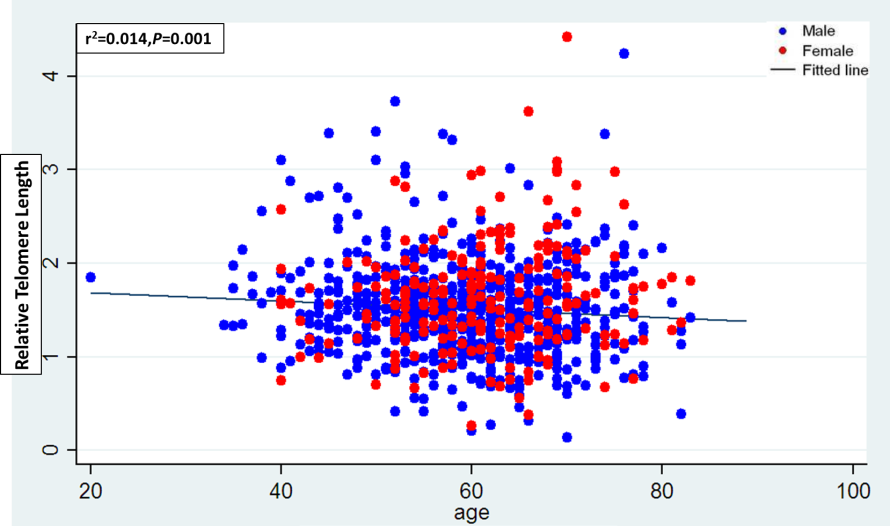


**Supplementary Figure 1:** Association between relative telomere length (RTL) and age among controls.

**Supplementary Figure 2-A:**

**
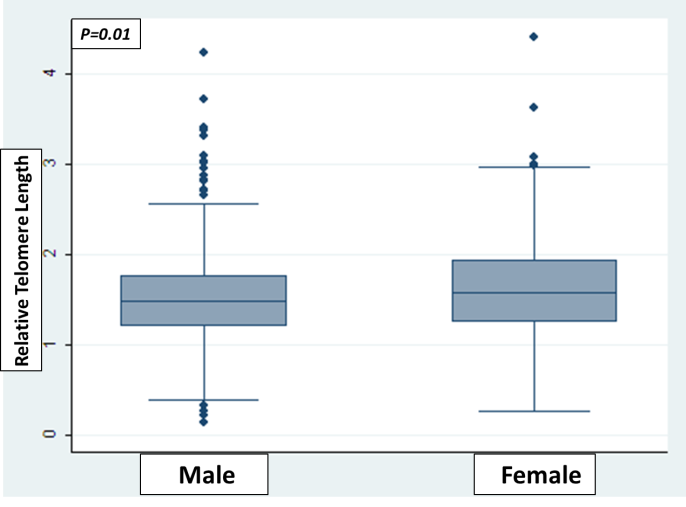
**

**Supplementary Figure 2-B:**


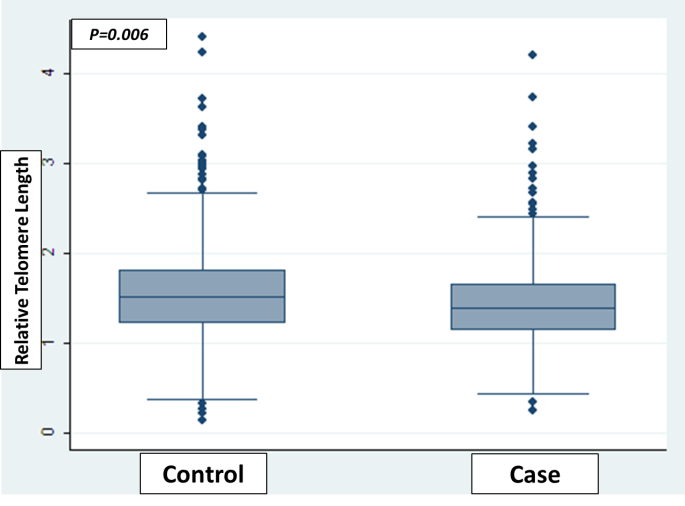


**Supplementary Figure2:** (2-A) Association of telomere length with gender among controls; (2-B) Association between relative telomere length (RTL) and SCCHN risk.

**Supplementary Table 1:** Stratified analyses of associations between relative telomere length (RTL) and SCCHN risk.

| Variable | RTL（Quartile classification） | | Adjusted OR (95%CI) | *P*^a^ | *P*^b^ |
| --- | --- | --- | --- | --- | --- |
|  | Controls(n=913) | Cases(n=510) |  |  |  |
| Age at diagnosis (years) |  |  |  |  |  |
| ＜60 | 141/117/104/71 | 52/55/71/53 | 1.29(1.11-1.50) | **0.001** | 0.231 |
| ≥60 | 88/111/124/157 | 46/43/86/104 | 1.14(1.00-1.31) | 0.054 |  |
| Sex |  |  |  |  |  |
| Male | 154/169/180/179 | 64/72/108/113 | 1.17(1.04-1.32) | **0.007** | 0.258 |
| Female | 75/59/48/49 | 34/26/49/44 | 1.33(1.10-1.60) | **0.002** |  |
| Smoking status^c^ |  |  |  |  |  |
| Never | 117/111/104/119 | 53/44/80/72 | 1.15(1.01-1.33) | **0.036** | 0.410 |
| Ever | 112/117/124/109 | 45/54/76/84 | 1.25(1.09-1.45) | **0.002** |  |
| Drinking status^c^ |  |  |  |  |  |
| Never | 150/150/133/141 | 57/45/77/76 | 1.18(1.04-1.35) | **0.012** | 0.806 |
| Ever | 79/78/95/87 | 41/53/79/81 | 1.21(1.04-1.41) | **0.013** |  |
| Tumor site |  |  |  |  |  |
| Oral | 229/228/228/228 | 69/77/125/132 | 1.28(1.14-1.43) | **<0.001** | **0.008** |
| Other sites | 229/228/228/228 | 29/21/32/25 | 0.96(0.80-1.15) | 0.636 |  |

^a^ Derived from additive model using logistic regression analysis with an adjustment for age, sex, smoking and drinking status.

^b^ *P* for heterogeneity test based on χ2-based Q test.

^c^ The smoking and drinking status were unavailable for two and one controls, respectively.

**Supplementary Table 2:** Stratified analyses of associations between rs2736100 and SCCHN risk.

| Variables | rs2736100 (*TERT*, TT/TG/GG) | | Adjusted OR (95%CI)^a^ | *P*^a^ | *P*^b^ |
| --- | --- | --- | --- | --- | --- |
|  | Controls(n=897) | SCCHNs(n=495) |  |  |  |
| Age (years) |  |  |  |  |  |
| < 60 | 141/205/80 | 65/109/47 | 1.13(0.90-1.42) | 0.292 | 0.669 |
| ≥ 60 | 176/214/81 | 79/146/49 | 1.21(0.97-1.49) | 0.083 |  |
| Sex |  |  |  |  |  |
| Male | 231/313/125 | 98/190/56 | 1.08(0.89-1.30) | 0.430 | 0.126 |
| Female | 86/106/36 | 46/65/40 | 1.41(1.06-1.87) | **0.019** |  |
| Smoking states^c^ | | | |  |  |
| Never | 154/217/73 | 70/118/56 | 1.29(1.03-1.61) | **0.026** | 0.240 |
| Ever | 163/202/88 | 73/136/40 | 1.07(0.86-1.33) | 0.522 |  |
| Drinking states^c^ | | | |  |  |
| Never | 207/262/95 | 70/127/51 | 1.28(1.03-1.58) | **0.023** | 0.182 |
| Ever | 110/157/66 | 74/127/45 | 1.03(0.81-1.30) | 0.809 |  |
| Tumor site |  |  |  |  |  |
| Oral | 317/419/161 | 111/194/85 | 1.24(1.05-1.46) | **0.013** | 0.094 |
| Other sites | 317/419/161 | 33/61/11 | 0.93(0.69-1.24) | 0.622 |  |

^a^ Derived from additive model using logistic regression analysis with an adjustment for age, sex, smoking and drinking status.

^b^ *P* for heterogeneity test based on χ^2^-based Q test.

^c^ The smoking and drinking status were unavailable for two and one controls, respectively.

**Supplementary Table 3:** Information of TL and SCCHN risk association studies included in the mini meta-analysis.

| Author | Tumor site | Study design | No. of controls/ cases | DNA source | Method of TL measurement | Adjust OR | References | Population |
| --- | --- | --- | --- | --- | --- | --- | --- | --- |
| Gu(Our study) | SCCHN | Case–control | 913/510 | PBLS | RT-PCR | 1.19(1.08-1.32) | Our study | Chinese |
| Wu(2003) | SCCHN | Case–control | 92/92 | PBLS | RT-PCR | 6.75(2.62-17.36) | 35 | American |
| Liu(2011) | SCCHN | Case–control | 885/888 | PBLS | RT-PCR | 0.97(0.80-1.17) | 32 | American |
| Bau(2013) | OSCC | Case–control | 394/92 | PBLS | RT-PCR | 3.47(1.86-6.53) | 34 | American |
| Zhang(2013) | OPC | Case–control | 335/188 | PBLS | RT-PCR | 1.70(1.10-2.60) | 36 | American |
| Zhang(2013) | OCC | Case–control | 335/137 | PBLS | RT-PCR | 0.80(0.50-1.20) | 36 | American |

Abbrevation: Squamous cell carcinoma of head and neck (SCCHN); Oral squamous cell carcinoma (OSCC); Oropharyngeal squamous cell carcinoma (OPC); Oral cavity cancer (OCC); Peripheral blood leukocytes (PBLs).

**Supplementary Table 4:** The primers and probes for q-PCR and selected genetic variants genotyping

| Locus | Primers (5'-3') | Probes |
| --- | --- | --- |
| TEL1 | GGTTTTTGA[GGGTGA]4GGGT |  |
| TEL2 | TCCCGACTAT[CCCTAT]4CCCTA |  |
| 36B4 | UP: CAGCAAGTGGGAAGGTGTAATCC |  |
|  | DOWN: CCCATTCTATCATCAACGGGTACAA |  |
| rs8105767 | F: TTGGCACCACATTCAGGTTATG | FAM-TAGCTGAGTCTGATACC-MGB |
|  | R: GGGCCAGGCCAGAGAGTTA | HEX-TGAGTCCGATACCCA-MGB |
| rs7675998 | F: AGTCCTGTCTTCTGAGTTTTAAGAATGC | FAM-ACATCAGTCATTTATTT-MGB |
|  | R: CTTCAGGCAGTTTTGAGCACAT | HEX-CACATCAGTTATTTATTT-MGB |
| rs755017 | F: GGAAGAATTGGCAGGACTCGTT | FAM-TCCTCCCGGCCAT-MGB |
|  | R: AGCAGCAGCACGGAAGCT | HEX-CTCCTCCCAGCCAT-MGB |
| rs4387287 | F: CACTTGTAGCCCCTCCCG | FAM-AGCCCTGCGACCTG-MGB |
|  | R: GACCTCAGCCAACTGCTCCT | HEX-AGCCCTGAGACCTG-MGB |
| rs2736100 | F: GACGGGGAACAAAGGAGGA | FAM-CAAAGCTAAAGAAACAC-MGB |
|  | R: GTTCTATCTCAGGCATCTTGACACC | HEX-CAAAGCTACAGAACA-MGB |
| rs11125529 | F: GAGAACAGGGACTATGTCTTACTCATCA | FAM-CTAAAACATCATCTTGGCC-MGB |
|  | R:TGTATTTTGAGCTTAGTTGTTTACAGATGTT | HEX-ACTAAAACATAATCTTGGCC-MGB |
| rs10936599 | F: CCGCTGTTTGTTCAGTCTCTCTAA | FAM-AGGTGCTCACAGTGG-MGB |
|  | R: GGTTACTGATTTACTGTTTCTTTTTGTCTTT | HEX-TGCTCACAATGGTG-MGB |
